# Single coronary ostium with obstructive hypertrophic cardiomyopathy treated using the Morrow procedure: a case report

**DOI:** 10.1186/s13019-022-02084-2

**Published:** 2022-12-28

**Authors:** Xin Lv, Jianhua Li, Chuanzhen Liu, Ruyuan Wei, Lingwei Meng, Xiangjin Kong, Kaiming Wei, Guangqing Cao, Kai Liu

**Affiliations:** 1 Department of Cardiovascular Surgery, Qilu Hospital of Shandong University, No.107, West Wenhua Road, Jinan, 250012 Shandong China; 2 Shandong University, Jinan, 250061 Shandong China; 3 Pantheum Biotechnology Co., Ltd, Shandong Jinan, China

**Keywords:** Single coronary artery, Hypertrophic obstructive cardiomyopathy, Morrow procedure, Cardiopulmonary bypass, Case report

## Abstract

**Background:**

Hypertrophic cardiomyopathy is a commonly inherited heart disease. In addition, single coronary artery (SCA) is a rare congenital anomaly of the coronary arteries. And SCA concomitant with severe hypertrophic obstructive cardiomyopathy (HOCM) has seldom been reported in the literature. However, such cases have not been reported to be treated with the Morrow procedure.

**Case presentation:**

Herein, we presented a case of a 64-year-old female diagnosed with a single left coronary artery with severe HOCM. The HOCM was treated with the Morrow procedure. The patient was discharged on the seventh postoperative day and was asymptomatic during the follow-up.

**Conclusion:**

To our knowledge, this is the first study reporting a single left coronary artery with severe HOCM treated with the Morrow procedure. In addition, myocardial protection by cardioplegia antegrade perfusion was safe for the patient with SCA and HOCM.

**Supplementary Information:**

The online version contains supplementary material available at 10.1186/s13019-022-02084-2.

## Introduction

Hypertrophic cardiomyopathy (HCM) is a commonly inherited heart disease affecting 1 in 200 people (0.5%) [1]. Furthermore, single coronary artery (SCA) is a rare congenital coronary anomaly. The incidence of SCA is 0.024–0.066% [2]. In 1979, Lipton et al. proposed a method for categorizing single coronary anomalies, which was further improved by Yamanaka and Hobbs in 1990 [3]. SCA is more frequent when it coexists with other congenital anomalies, however, it could also present as isolated congenital heart disease [3]. In addition, several cases of HCM coexisting with abnormal coronary origin have been reported [4–6]. However, severe hypertrophic obstructive cardiomyopathy (HOCM) with SCA treated with the Morrow procedure is not reported in the literature. Our current case report might provide a basis for the treatment of patients with coexisting HOCM and SCA.

## Case presentation

**Fig. 1:**
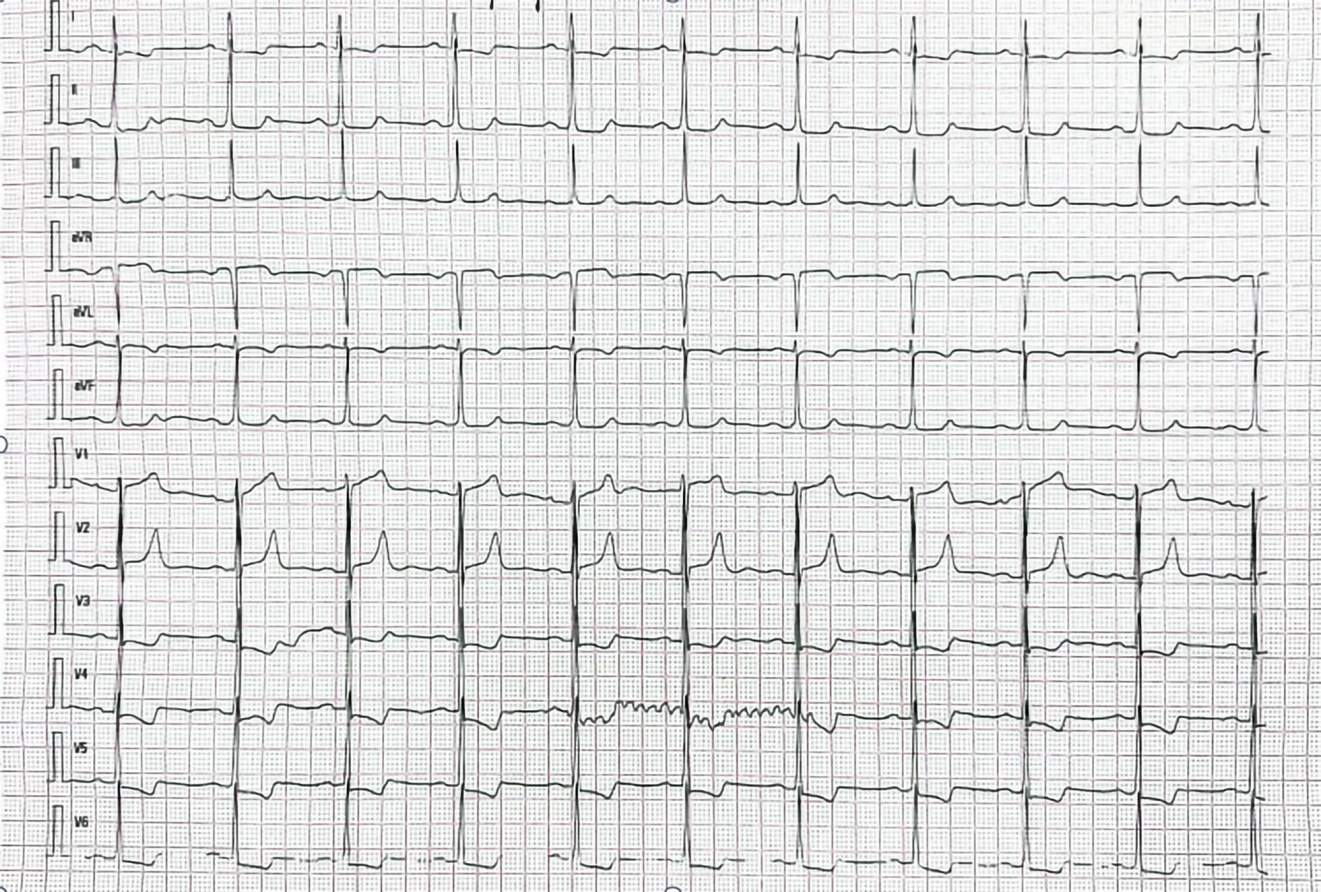
The electrocardiogram preoperatively

**Fig. 2:**
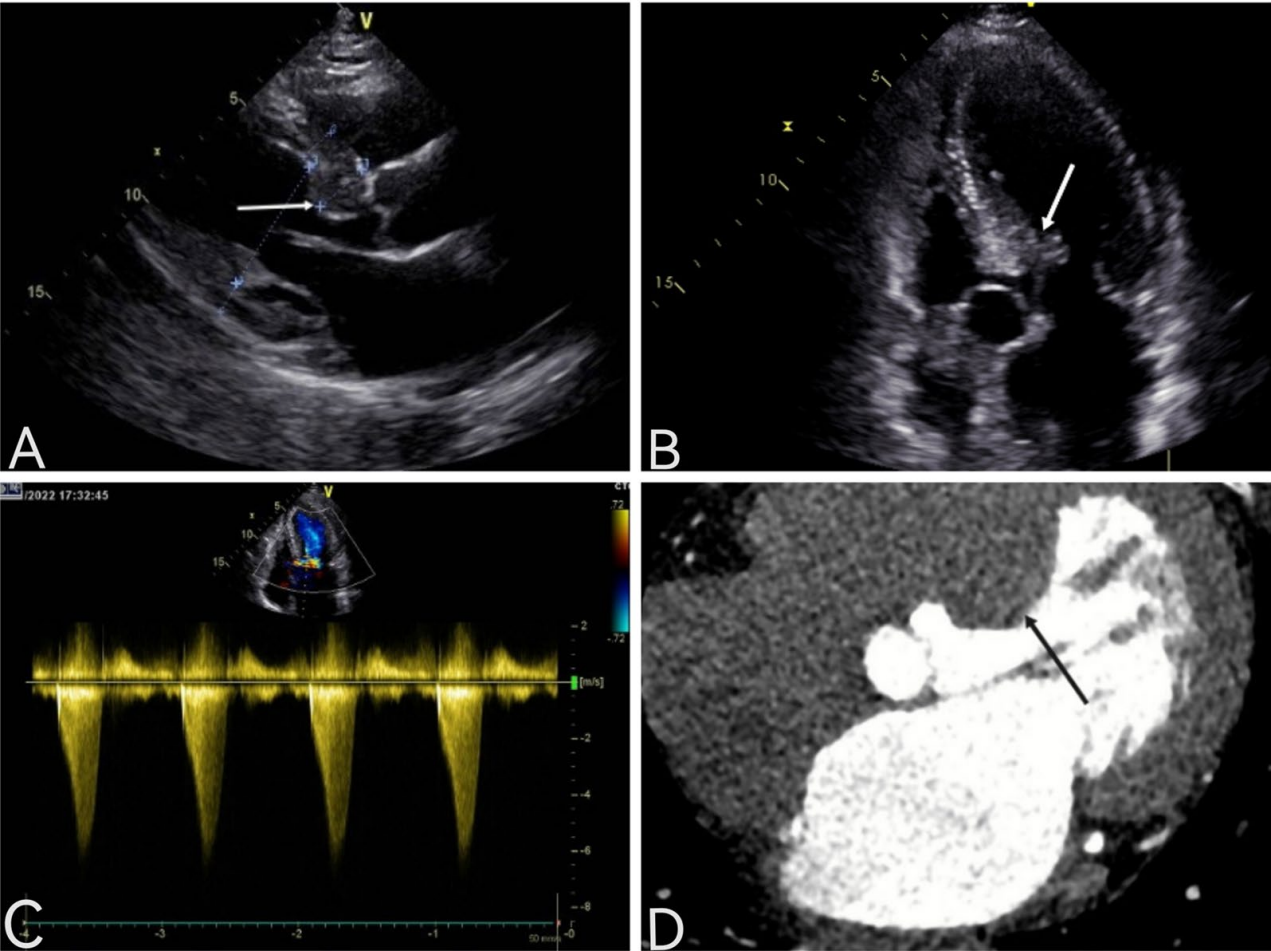
Echocardiography preoperativelythe basal part of the interventricular septum (IVS) was thickened (**A**, arrow).SAM was obvious (**B**, arrow).Systolic blood flow image in a specific direction from the left ventricle to the aorta (**C**, arrow).Coronary CTA showing the hypertrophic myocardium clearly protrudes into the left ventricular outflow tract (LVOT) (**D**, arrow)

**Fig. 3:**
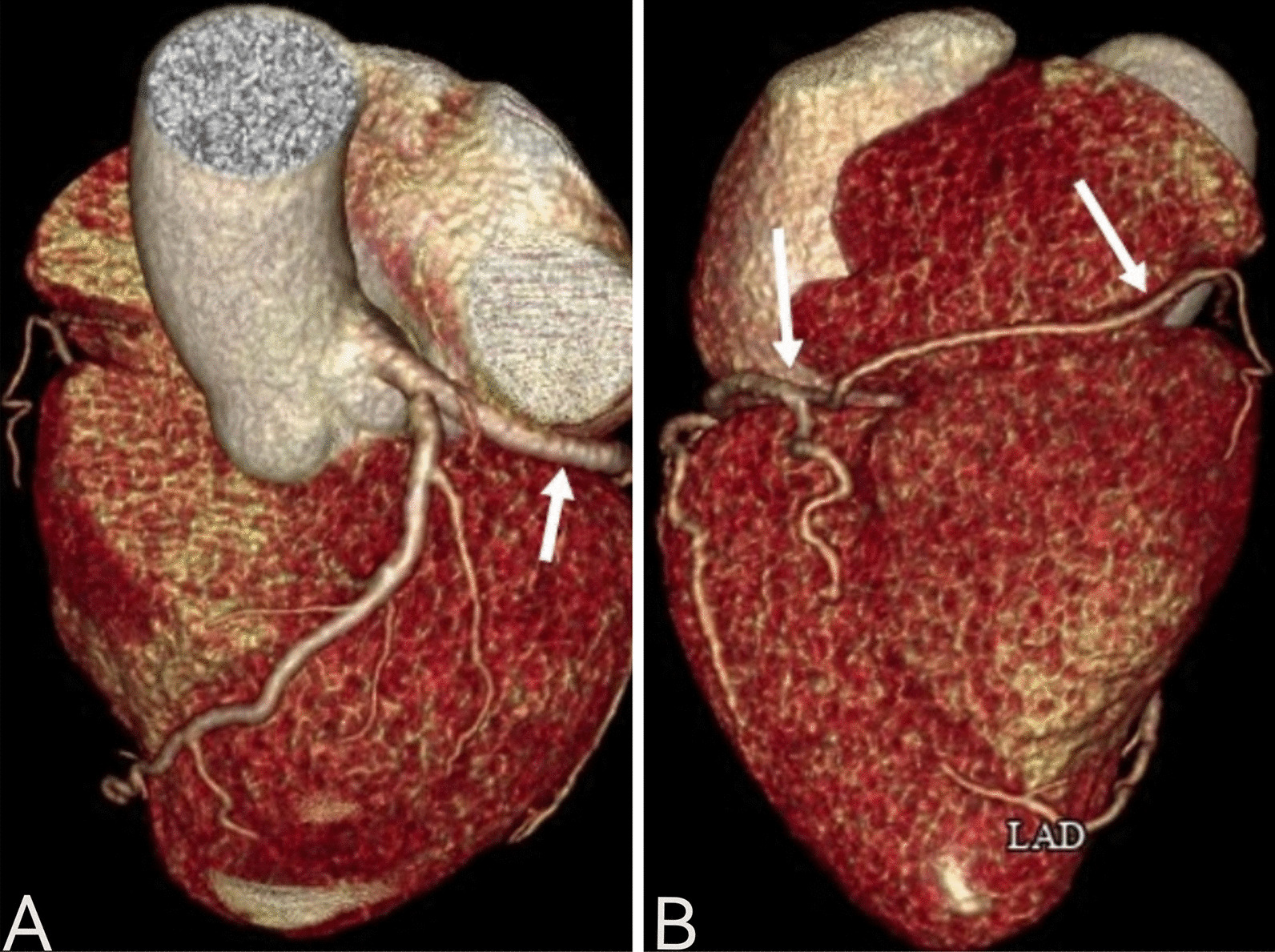
Three-dimensional coronary artery CTA imaging showing the abnormal origin and course of the coronary artery.The giant circumflex (CX) branch of the LCA supplied the right coronary artery blood supply area (**A** and** B**, arrow)

**Fig. 4:**
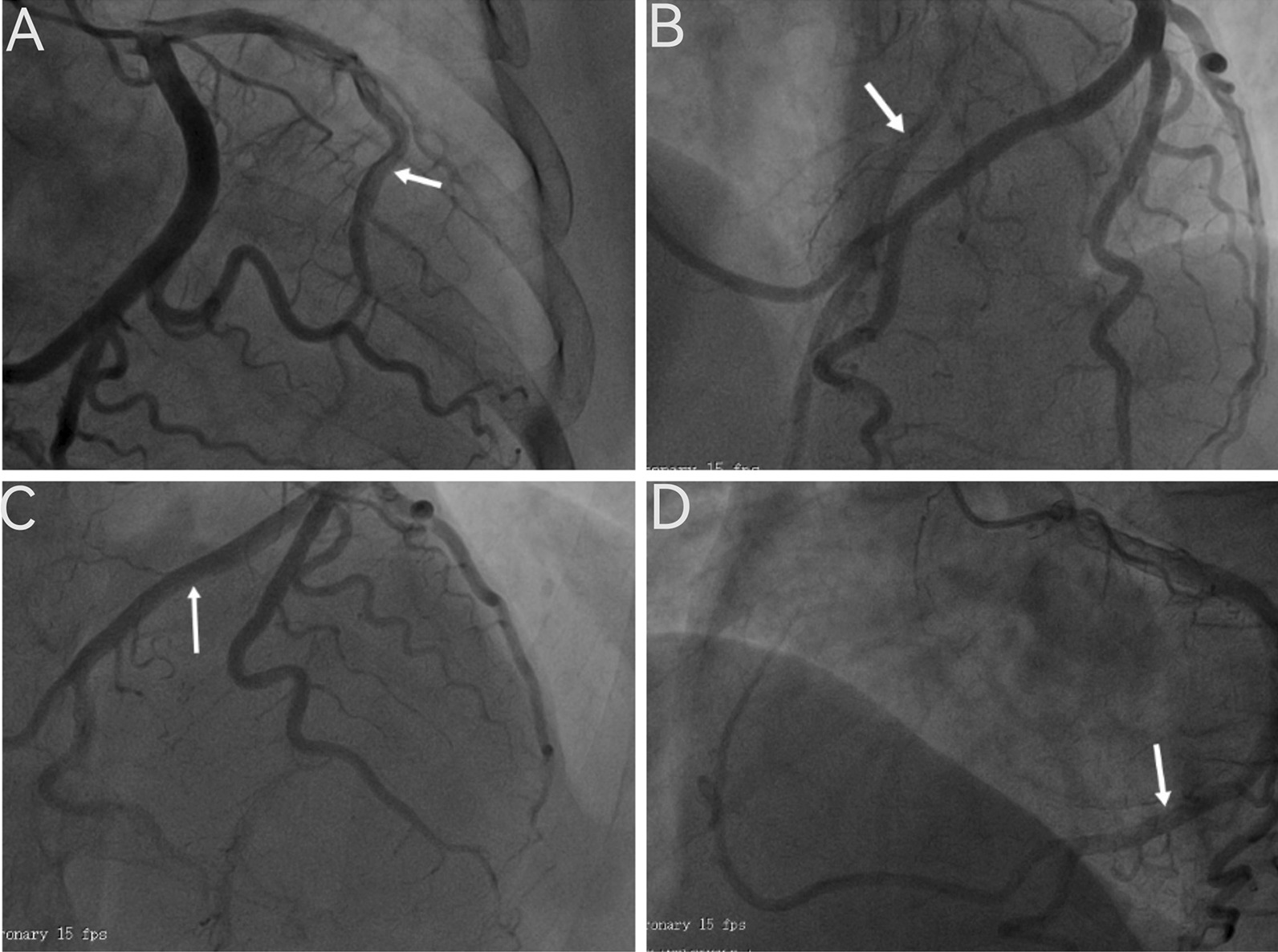
Coronary angiography (DSA) showing a single coronary artery(SCA).Anterior descending artery (LAD) (**A** and** B**, arrow).The giant circumflex (CX) branch of the LCA (**C** and** D**, arrow)

**Fig. 5:**
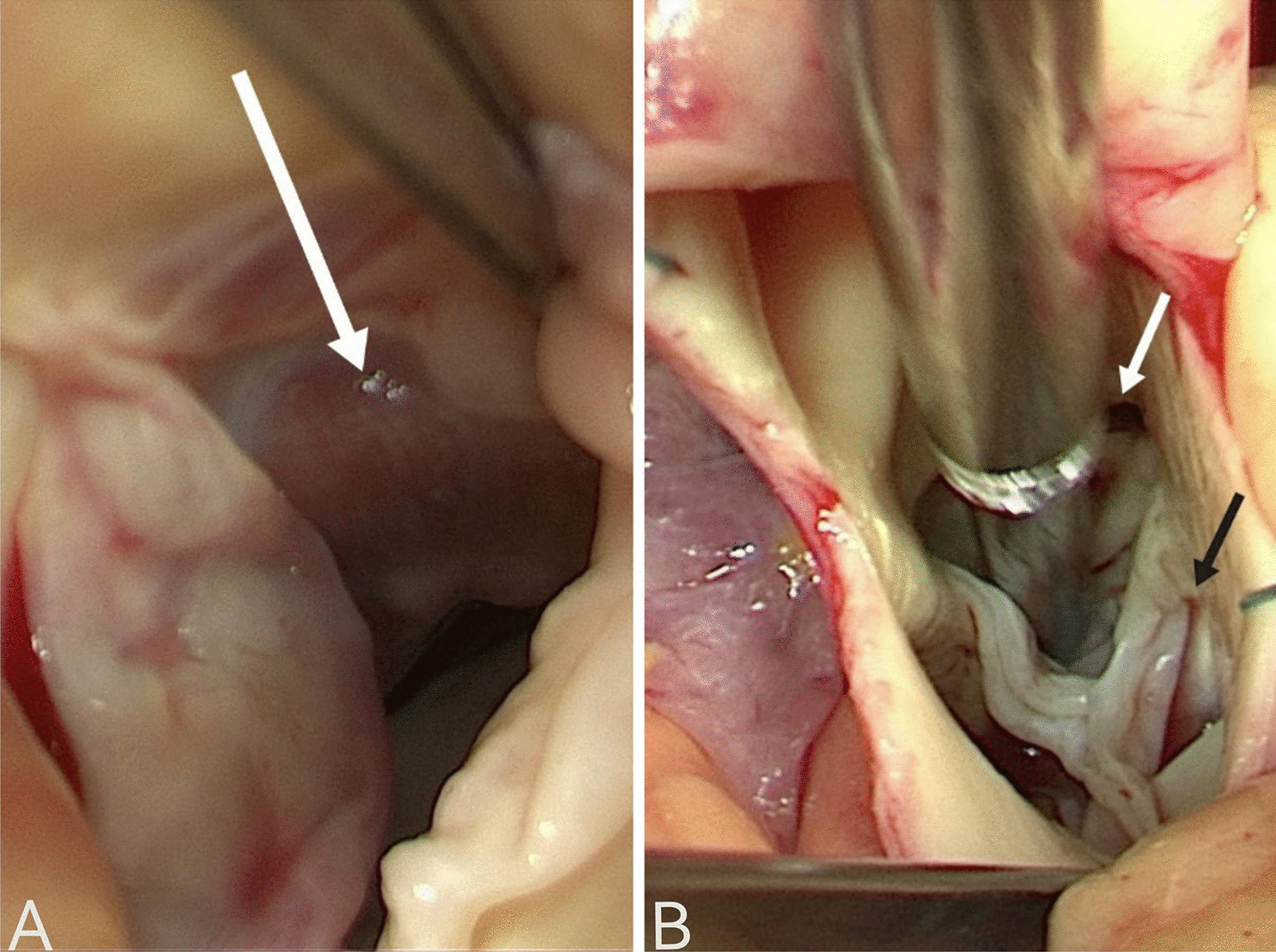
Imaging of the operation.The hypertrophic myocardium of the basal part of the IVS (**A**, arrow).A single left coronary ostium (**B**, white arrow).There is no right coronary artery opening in the right sinus of aortic sinus (**B**, black arrow)

**Fig. 6:**
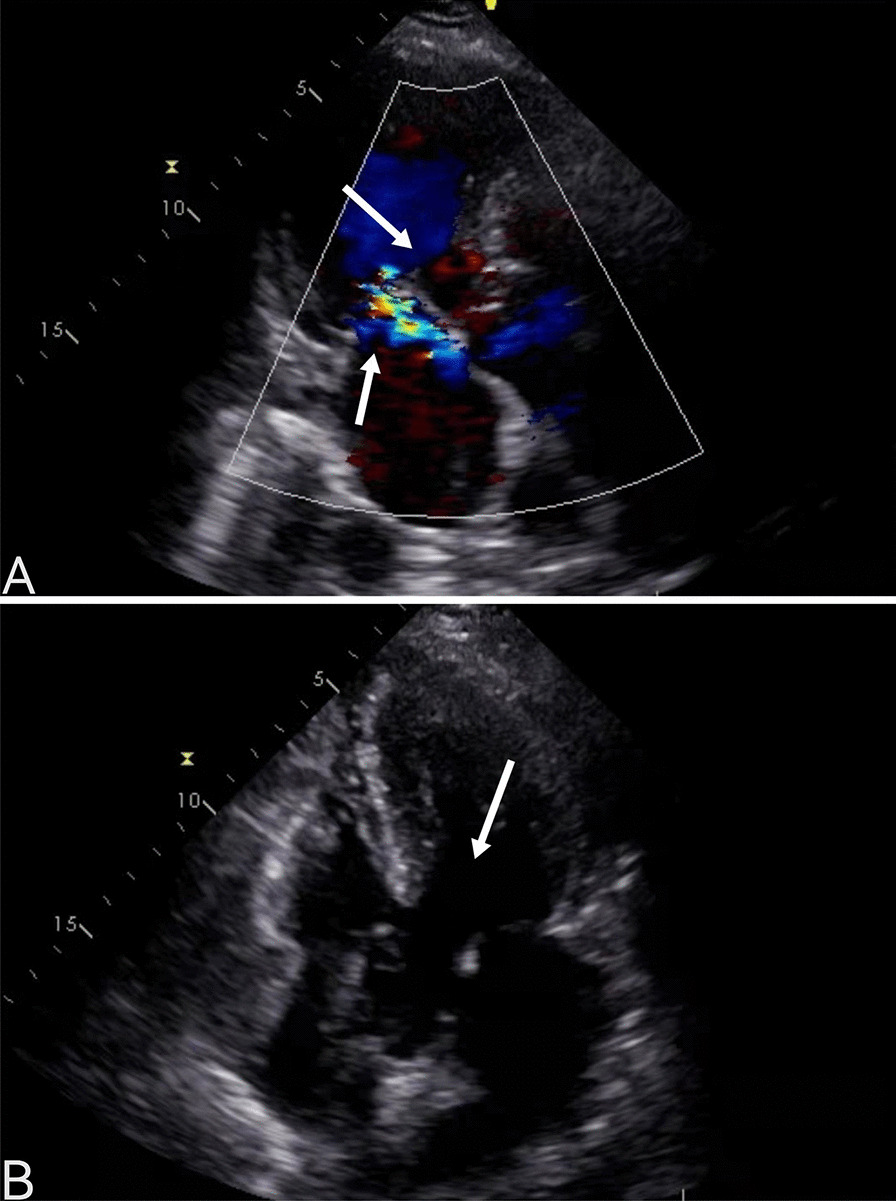
Echocardiography postoperatively.The mitral valve regurgitation was mild (**A**, the arrow below). LVOT blood flow is unobstructed (**A**, The arrow above). The SAM sign disappeared (**B**, arrow)

A 64-year-old woman was admitted to the hospital because of chest tightness and shortness of breath. The physical examination revealed a grade III/VI systolic murmur with tremors in the third intercostal space of the left sternal border. The patient had no other significant clinical manifestations. A family history of hypertension was reported upon inquiry (her father and two older sisters had hypertension) and our patient suffered from grade II hypertension (very high risk). The patient underwent left upper lobectomy for lung cancer 5 years ago, and the postoperative recovery was satisfactory. Holter electrocardiogram showed occasional atrial premature beats, frequent multi-source premature ventricular contractions, and ST-T changes (Fig. 1). Echocardiography showed that the left ventricular ejection fraction (LVEF) was 63%, and the basal part of the interventricular septum (IVS) was thickened (diastole, 20 mm). The hypertrophic myocardium protruded into the left ventricular outflow tract (LVOT) (diastole, Fig. 2A), which caused a severe obstruction of LVOT. The mitral valve regurgitation (severe) and systolic anterior motion (SAM) were observed in the systolic stage (Fig. 2B). In addition, the diastolic function of the left ventricle was reduced (Additional file 1: Movie 1). The echocardiograph showed that the systolic blood flow was in a specific direction from the left ventricle to the aorta with a peak velocity of 6.08 m/s, maximum transvalvular pressure of 147.73 mm Hg, and a mean pressure of 63.37 mm Hg (systole, Fig. 2C, Additional file 2: Movie 2). Coronary computed tomography angiography (CTA) showed significant stenosis of the LVOT (Fig. 2D), and the right coronary artery (RCA) was absent. Three-dimensional (3D) -CTA imaging revealed the abnormal origin and course of the coronary artery (Fig. 3A, B). The subsequent conventional coronary angiography (CCA) examination further confirmed the diagnosis of SCA. The left coronary artery (LCA) originated from the left aortic sinus and spread throughout the heart. The great circumflex (CX) branch of the LCA supplied the area that the RCA supplies in the normal condition (Fig. 4). The patient was administered beta-blockers and calcium antagonists before admission but they had no significant effect. The patient had severe hypertrophy of the basal part of IVS, combined with SCA, and it was difficult to locate the target vessel, so the expected effect of alcohol radiofrequency ablation was poor. After the comprehensive evaluation of the preoperative condition and exclusion of the surgical contraindications, the Morrow procedure was performed under cardiopulmonary bypass (CPB). Anterograde perfusion of the aortic root was adopted during the surgery, and cardiac arrest was successfully induced. The intraoperative findings were consistent with the preoperative data: the myocardium with obvious hypertrophy of the basal part of the IVS protruded into the LVOT (Fig. 5A), and RCA could not be detected (Fig. 5B). During the surgery, the hypertrophic myocardium was removed carefully, which unclogged the LVOT. About 3 × 2 × 0.8 cm^3^ hypertrophic myocardium was cut longitudinally from 8 to 10 mm below the midpoint of the inferior margin of the right coronary valve to the junction of the left and right coronary valves. Further exploration showed that the mitral valve and subvalvular apparatus were normal. The patient was discharged on the seventh postoperative day. One day before discharge, the echocardiograph showed that the maximum transvalvular pressure between the LVOT and the aorta was 15 mm Hg, the mitral valve regurgitation was mild (Fig. 6A), the LVOT had been dredged (Fig. 6B), and the SAM had disappeared (Additional file 3: Movie 3). After more than 3 months of follow-up, the patient was asymptomatic.

## Discussion and conclusions

HCM is a commonly inherited heart disease with an incidence of about 0.5% [1]. The clinical presentation of HCM is diverse and the pathophysiology is complex, yet therapeutic strategies are available and effective. HCM has transitioned from a rare and nearly incurable disease to a common genetic disorder [7]. It is considered an important cause of arrhythmic cardiac arrest, heart failure, and atrial fibrillation (with embolic stroke) [8]. Clinical diagnosis is primarily based on unexplained left ventricular hypertrophy as determined using echocardiography or cardiovascular magnetic resonance imaging (MRI) [9].

SCA is a rare congenital developmental abnormality of the coronary artery with an incidence of 0.024–0.066%, where only one coronary artery emerges from the coronary ostium and supplies the entire heart [2]. In 1979, Lipton et al. proposed a classification of SCA malformations based on a large number of cases. The classification was further improved by Yamanaka and Hobbs in 1990, who divided it into different subtypes according to the origin and course of the coronary artery [3]. The first level of classification is right (R) and left (L) types, according to the origin of the SCA; the second level of classification is types I, II, and III, according to the course of the coronary branches. In type I, the artery runs along the anatomical route of the LCA or the RCA. The CX provides the posterior descending artery and runs in the posterior atrioventricular groove, and the CX branches supply the right atrium and the right ventricle; in the absence of the LCA, a large RCA is observed in the posterior atrioventricular groove, which extends to the anterior base of the heart, where it forms the left anterior descending artery (LAD). In type II, the other coronary artery originates proximal to the normally located one and passes through the base of the heart before reaching the normal part of the native coronary artery. In type III, the SCA originates from the right coronary sinus, while the LAD and left circumflex (LCX) branches originate from the common trunk. According to this classification, our patient belonged to the L-I type originating from the left coronary sinus.

HCM and coronary artery abnormalities are considered to be the leading causes of exercise-related cardiac arrest, especially in young adults [10]. Although SCA can occur as isolated congenital heart disease, its incidence increases when it coexists with certain other congenital anomalies. Congenital anomalies coexisting with SCA include coronary aneurysm [11], coronary arteriovenous fistula [12], ventricular septal defect [13], transposition of great vessels [14], patent foramen ovale [15], tetralogy of Fallot [16], trunk arteriosus [17], patent ductus arteriosus [18], and bicuspid aortic valve [19]. In addition, several cases of HCM coexisting with abnormal coronary origin have been reported [4–6]. Although cases of HCM with SCA are reported [20], ours is the first report where such a case was treated with the Morrow procedure. The current report suggests that the Morrow procedure is an effective treatment of HOCM with SCA.

The treatment of HOCM is mainly to relieve symptoms and prevent sudden death. For symptomatic patients, negative inotropic drugs including beta-blockers and calcium antagonists can relieve symptoms, while for drug-refractory patients, surgical treatment, including Alcohol septal ablation (ASA) and myectomy, should be considered [21]. Our patient was administered beta-blockers and calcium antagonists before admission but they had no significant effect. The LVOT differential pressure was large, and the patient was symptomatic. Studies have shown that Cibenzoline treatment significantly reduces all cardiovascular complications and death due to left ventricular heart failure and may be a promising treatment for HOCM patients [22]. ASA is an effective method for the treatment of HOCM, and its safety and efficacy have been confirmed by several studies; however, certain patients, especially those with severe basal thickening, do not benefit from the treatment. The patient had severe hypertrophy of the basal part of IVS, combined with SCA, and it was difficult to locate the target vessel, so the expected effect of ASA was poor. Furthermore, HCM patients with left ventricular systolic dysfunction (defined as left ventricular ejection fraction < 50%) have a poor prognosis [23]. The LVEF of our patient was 0.63, and after the comprehensive evaluation of the preoperative condition and exclusion of the surgical contraindications, the Morrow procedure under CPB was identified as the best choice. During the Morrow procedure, the extent of resection of hypertrophic myocardium should be determined according to the actual situation observed during the surgery, not only to relieve the SAM but also to avoid serious complications such as third-degree conduction block and left ventricular rupture caused by excessive myocardial resection.

Various rare courses of SCA can lead to accidental ligation or damage to important blood vessels during cardiac surgery. Therefore, the cardiac surgeon and coronary angiographer should be familiar with the presence and anatomy of this congenital anomaly. Because of the rarity of this condition and the complications during surgery, it is recommended that CCA should be performed before cardiac surgery. In addition, SCA should also be paid attention to during cardiopulmonary bypass surgery, because the effect of myocardial protection was related to the strategy of cardioplegia perfusion in such patients. In our case, the CCA and echocardiographic imaging data of the patient were discussed in detail before the surgery, which suggested that there was no obvious abnormality of the aortic valve. It was agreed that anterograde perfusion in the aortic root would be safe and effective, and direct perfusion of the SCA would be performed if necessary. The current report is only for individual cases, patients with the same disease need individual solutions.

## Supplementary Information

**Additional file 1: Movie 1.** Preoperative Echocardiography showed the LVOT was severely obstructed, and the SAM was obvious

**Additional file 2: Movie 2.** Preoperative Echocardiography showed systolic blood flow accelerated in a specific direction from the left ventricle to the aorta and the severe mitral valve regurgitation

**Additional file 3: Movie 3.** Postoperative echocardiography showed LVOT blood flow is unobstructed and the SAM disappeared

## Data Availability

As this paper is a case report, all data generated or analysed are included in this article.

## Abbreviations

HCM: Hypertrophic cardiomyopathy
SCA: Single coronary artery
HOCM: Hypertrophic obstructive cardiomyopathy
LVEF: Left ventricular ejection fraction
IVS: The interventricular septum
LVOT: Left ventricular outflow tract
SAM: Systolic anterior motion
CTA: Computed tomography angiography
RCA: Right coronary artery
CCA: Conventional coronary angiography
LCA: Left coronary artery
CX: Circumflex
CPB: Cardiopulmonary bypass
LAD: Anterior descending artery
LCX: Left circumflex
ASA: Alcohol septal ablation
